# The value of time savings in breast cancer therapy for patients and healthcare providers: the subcutaneous injection of pertuzumab and trastuzumab fixed-dose combination at a tertiary care center in the UAE

**DOI:** 10.57264/cer-2026-0007

**Published:** 2026-06-02

**Authors:** Khaled H.A. Al Qawasmeh

**Affiliations:** 1 Senior Charge Nurse, Tawam Hospital, Abu Dhabi, UAE

**Keywords:** breast cancer, fixed-dose combination, pertuzumab, subcutaneous, switching, time saving, trastuzumab

## Abstract

**Aim:**

To measure time saving associated with the subcutaneous (SC) fixed-dose combination of pertuzumab and trastuzumab (PH FDC SC) compared with intravenous (IV) pertuzumab and trastuzumab in patients with HER2-positive breast cancer.

**Materials & methods:**

This was a single-center local observational study consisting of a retrospective chart review of patients who received PH FDC SC or IV pertuzumab and trastuzumab in the UAE. Eligible patients were adults aged ≥18 years diagnosed with early or metastatic breast cancer. Data collected included demographics, treatment indication and nursing documentation of medication administration. The primary end point was a composite of patient length of stay during administration of SC versus IV pertuzumab and trastuzumab, and pharmacist preparation time. For secondary end points, annual clinical hours saved were extrapolated based on a 100% switching rate for a cohort of 75 patients undergoing 18 cycles annually (1350 visits).

**Results:**

Fifty patients with three visits per patient were included in the final analysis; PH FDC SC was administered in 50% of the visits resulted in a total of 150 occurrences of treatment administration. The mean age was 47.0 ± 10.9 years. Patients' length of stay was significantly shorter in the SC arm by a mean difference of 190.9 ± 13.6 min saved per patient (95% CI: 163.9, 217.9). Median pharmacist preparation time was significantly shorter (8 min [Q1: 4; Q3: 15]; p = 0.006) with the SC formulation compared with the IV formulation (13 min [Q1: 6; Q3: 28]). When extrapolated, switching to the SC formulation would save 2468.25 clinical hours saved annually across nursing and pharmacy services.

**Conclusion:**

PH FDC SC was associated with significant time savings for both patients and healthcare providers. The transition to SC administration unlocked 2468.25 clinical hours annually, translating into notable indirect cost reductions through optimized resource utilization and improved operational efficiency compared with IV administration.

## Plain language summary

**What is this summary about?:**

The combination of pertuzumab and trastuzumab was made available as a medication for breast cancer to be given under the skin. This product was equally effective and safe as that given directly into bloodstream. The under-skin form was more convenient for physicians and patients in terms of shorter time needed to receive the medication. The aim of this local study was to measure the time saved by breast cancer patients when the combined medication was given under the skin compared with that given directly into a vein. Another aim was to measure time spent by pharmacists for preparing each formulation.

**What were the results?:**

Fifty patients with three visits per patient were included. Patients' length of stay was significantly shorter in the under-skin group by 190.9 min saved per patient. Similarly, pharmacy preparation time was significantly shorter (8 vs 13 min) with the under-arm formulation compared with the formulation given into the vein.

**What do the results of the studies mean?:**

The under-arm form of the combination of pertuzumab and trastuzumab was associated with significant time savings for both patients and healthcare providers compared with the form given directly through the vein.

The subcutaneous (SC) formulation of a new fixed-dose combination (FDC) of pertuzumab, trastuzumab and hyaluronidase–zzxf was approved by the US FDA on 29 June 2020 [1]. In the UAE, SC FDC of pertuzumab and trastuzumab (PH FDC SC) is locally approved for both early breast cancer and metastatic breast cancer according to the regional prescribing information [2]. Specifically, it is indicated for the neoadjuvant treatment of adult patients with human epidermal growth factor receptor 2-positive (HER2), locally advanced, inflammatory, or early-stage breast cancer at high risk of recurrence; and for the adjuvant treatment of adult patients with HER2-positive early breast cancer at high risk of recurrence. Furthermore, it is approved in combination with docetaxel for adult patients with HER2-positive metastatic or locally recurrent unresectable breast cancer who have not received prior anti-HER2 therapy or chemotherapy for metastatic disease.

Studies have shown that the PH FDC SC is as safe and effective as the intravenous (IV) formulation. The Phase III FeDeriCa study demonstrated that PH FDC SC was noninferior to IV pertuzumab plus trastuzumab in terms of serum levels in patients with HER2-positive early breast cancer [3]. Similarly, the PHranceSCa study emphasized the long-term clinical efficacy and safety of PH FDC SC besides improving health-related quality of life [4].

PH FDC SC has identical active ingredients to pertuzumab and trastuzumab, containing 1200 mg pertuzumab plus 600 mg trastuzumab in a fixed, nonweight-based loading dose of 15 ml as a starting dose, and fixed, nonweight-based maintenance doses of 600 mg pertuzumab plus 600 mg trastuzumab in 10 ml. PH FDC SC also contains 2000 U/ml of the permeation enhancer, recombinant human hyaluronidase [3].

The SC formulation of PH FDC SC allows patients to receive treatment in 5–8 min [5,6] compared with approximately 30–90 min with the IV formulation [7]. Patients will be monitored for approximately 15–30 min after the SC administration [5,6] while patients have to wait for 30–60 min after the infusion to check for any infusion-related or allergic reaction [7].

The nonclinical advantages of SC administration for oncology biologics as a class have been highlighted in several studies. A systematic review of oncology biologics showed that the SC administration of oncology biologics was associated with reduced healthcare providers time and patient hospital time. In addition, resources use including non-drug consumables and drug wastage were also reduced. An additional cost saving was reported due to reduced productivity and leisure time loss [8]. Building on these general class benefits, a specific cost minimization analysis of PH FDC SC also showed that switching to PH FDC SC has led to significant monetary and non-monetary savings for the healthcare system and patients [9]. Furthermore, the primary analysis of the randomized, crossover of the PHranceSCa study reported that PH FDC SC was strongly preferred by most of the patients (85%; 136/160) and healthcare providers over intravenous administration after experiencing three cycles of each treatment route [10]. This preference was attributed to reduced resources utilization, time-saving benefits and greater patient convenience, without compromising healthcare providers interaction time [4,10]. Tawam Hospital Oncology Center’s experience with SC trastuzumab was favorable in terms of reducing healthcare worker time and save chair time in the clinic compared with IV infusions. The same benefits with PH FDC SC to patients and healthcare providers can be suggested. With the new formulation, PH FDC SC could save patient and healthcare provider time given its shorter injection time and observation time compared with IV infusions. Consequently, it would contribute to higher productivity and increased in capacity to serve more patients, keeping in mind cost reduction to healthcare facilities [11].

In this context, the objective of this observational retrospective study was to measure time saving associated with using PH FDC SC compared with pertuzumab and trastuzumab for IV infusion in patients with HER2-positive breast cancer, and its impact on indirect cost saving with higher productivity achieved by freeing more time to accommodate more patients in the service.

## Materials & methods

### Study design

This was a local observational study consisting of retrospective charts review of all patients who received PH FDC SC or pertuzumab and trastuzumab for IV infusion in their approved indications between 1 January 2020 and 30 December 2023 at Tawam Hospital Oncology Center, Al Ain in the UAE.

### Selection criteria

Eligible patients were adults aged ≥18 years diagnosed with early or metastatic breast cancer who received at least one dose of PH FDC SC or pertuzumab and trastuzumab for IV infusion in their approved indications between 1 January 2020 and 30 December 2023 at Tawam Hospital Oncology center, Al Ain, UAE.

Early breast cancer patients were either those diagnosed with HER2-positive, locally advanced, inflammatory, or early-stage breast cancer (either greater than 2 cm in diameter or node positive) at high risk of recurrence, receiving neoadjuvant therapy, or those at high risk of recurrence receiving adjuvant treatment; or diagnosed with HER2-positive early breast cancer at high risk of recurrence receiving adjuvant treatment of PH FDC SC or pertuzumab and trastuzumab for IV infusion. Metastatic breast cancer patients were those diagnosed with HER2-positive metastatic or locally recurrent unresectable breast cancer, who have not received previous anti-HER2 therapy or chemotherapy for their metastatic disease. Patients without any assessable medical records, or those receiving PH FDC SC or IV pertuzumab and trastuzumab IV infusion as part of any off-label combinations or indications were excluded.

### Data collection

Retrospective chart review was done by oncology professional nurses who extracted data from the center’s electronic health record system (Cerner Millennium HIM). This system provided precise, real-time documentation of the nursing workflow, including the initiation and completion of pre-medication, medication administration and post-treatment observation. The following data were collected and recorded on a standardized, anonymous electronic case report form: demographics, treatment indication (diagnosis, stage of disease and hormonal status), nursing documentation of the administration of PH FDC SC or pertuzumab and trastuzumab for IV infusion including the dose, medication orders in electronic medical records, pharmacy preparation duration, treatment duration, monitoring duration, nurse administration duration, patient length of stay and chair occupancy duration. Data were cross-referenced between the automated timestamps of the center’s electronic health record system and the nurses’ medication recorded time data log sheets to ensure documentation consistency and clinical accuracy.

### End points

The primary end point was composite consisting of patient length of stay during treatment and pharmacist preparation time. Patient length of stay during administration of PH FDC SC versus pertuzumab and trastuzumab for IV infusion was defined as the total duration from the start of pre-medication or clinical preparation to the completion of the post-administration observation period. Pharmacist preparation time was defined as the active time required for reconstituting and/or preparing the SC and IV formulations. The secondary end points included cost savings derived from time savings based on assumed hourly rates of pharmacists and nurses, in addition to chair occupancy duration, duration of nurse administration of both SC and IV formulations, and monitoring duration after giving both formulations. Chair occupancy duration was defined as the physical time a patient spent in the treatment chair during the clinical encounter. Nurse administration duration was defined as the specific, recorded time spent performing the physical administration (the SC injection or the IV infusion) and the immediate clinical checks associated with that task. The mean nurse time saved was then derived by calculating the difference between the IV and SC formulations across the entire patient encounter (preparation, administration and monitoring) per patient visit. Based on the observed time savings in pharmacist preparation and nurse administration, the total annual saved clinical hours were calculated by multiplying the mean saved minutes per visit by 1350 annual visits, considering a cohort of 75 patients undergoing 18 cycles per year (calculated as 365 days/21-day cycle). Potential annual indirect cost savings were then estimated by multiplying these total saved hours by assumed hourly labor rates (50–100 AED, equivalent to 13.6–27.3 USD for nurses, and 100–150 AED, equivalent to 27.3–40.9 USD for pharmacists, at the fixed conversion rate of 1 USD = 3.67 AED).

### Sample size

All available medical records for eligible patients during the study duration were reviewed to be included in this analysis. It was estimated that 150 independent treatment occurrences (50 patients with three independent treatment occurrences each) had to be included. Specifically, each study arm consisted of 25 patients with three independent treatment occurrences each, resulting in 75 independent treatment occurrences per arm.

With 75 independent treatment occurrences in each study arm, the study was able to detect a moderate effect size difference in the primary outcome of time spent in administering the SC injection and IV formulations between the two arms using an independent *t*-test with a power of 80% and a significance level of 5%. Moreover, with 75 independent treatment occurrences in each group, the study was able to estimate the time saving (difference in time between the two study arms) to within a margin of error of 0.32 standard deviation (SD) using 95% CI.

### Statistical analysis

Data were summarized using descriptive statistics. Continuous variables were reported as mean and SD when normally distributed and as median and interquartile range when skewed. Categorical data were summarized as frequencies and percentages. The normality of the data was tested visually using Q–Q plots and histograms, and statistically using the Kolmogorov–Smirnov test. There was no imputation for missing data.

Numeric variables were compared between the two study arms using the Student’s *t*-test when data were normally distributed, and the Mann–Whitney U test when showed a skewed distribution in at least one of the compared groups. In addition, mean and standard error of the differences were presented along with their 95% CIs. Categorical variables were compared between the two study arms using the Chi-square test, or Fisher’s exact test when expected counts were <5. Differences were regarded as statistically significant if the p-value was ≤0.05. Data were analyzed using the IBM Statistical Package for the Social Sciences for Windows release (IBM SPSS, version 29.0).

### Ethical considerations

This retrospective study was conducted in accordance with the Declaration of Helsinki, the study protocol, and in compliance with the applicable regulatory authority requirements in the participating site. It was also conducted in accordance with scientific purpose, value, and rigor, and follow generally accepted research practices such as Good Pharmacoepidemiology Practices issued by the International Society for Pharmacoepidemiology. The study protocol was approved by the Institutional Review Board/Independent Ethics Committee (IRB/IEC) of the participating institution. A documented waiver of the informed consent was granted in accordance with local regulatory authorities and IRB/IEC.

## Results

### Baseline characteristics

A total of 50 female patients were included in the final analysis, each completing three treatment visits, yielding a total of 150 independent treatment occurrences. The mean age of the patients was 47.0 ± 10.9 years and ranged between 26 and 74 years. The most represented nationality was Emirati (42%; n = 21), followed by Filipino (12%; n = 6) and Indian (8%; n = 4; Table 1).

**Table 1.** Patients’ demographics (N = 50).

Variable		
Age (years)	Mean ± SD	47.0 ± 10.9
	Median (Q1 – Q3)	46.5 (38.5–56.0)
	Min – max	26–74
Gender, n (%)	Female	50 (100.0%)
	Male	0 (0.0%)
Nationality, n (%)	Emirati	21 (42.0%)
	Filipino	6 (12.0%)
	Indian	4 (8.0%)
	Jordanian	3 (6.0%)
	Syrian	3 (6.0%)
	Saudi	2 (4.0%)
	Egyptian	2 (4.0%)
	Pakistani	2 (4.0%)
	Uzbekistani	2 (4.0%)
	Palestinian	1 (2.0%)
	Iraqi	1 (2.0%)
	Yemeni	1 (2.0%)
	Bangladeshi	1 (2.0%)
	Tanzanian	1 (2.0%)

SD: Standard deviation.

Across the 150 visits, IV pertuzumab and trastuzumab were administered in 50% of the visits while the remaining 50% received PH FDC SC). All IV infusions and SC injections were maintenance doses (IV: 600 mg pertuzumab plus 600 mg trastuzumab in 10 ml; infusions: 420 mg pertuzumab plus 600 mg trastuzumab). Most patients in both treatment arms (76% in the IV arm and 88% in the SC arm) were treated for HER2-positive metastatic or locally recurrent unresectable breast cancer, without prior exposure to anti-HER2 therapy or chemotherapy for their metastatic disease (Table 2).

**Table 2.** Treatment and indication.

Variable		Fixed-dose combination of pertuzumab and trastuzumab for subcutaneous injection, n (%)	Pertuzumab and trastuzumab for intravenous infusion, n (%)
		75 (50.0%)	75 (50.0%)
Dose	Loading dose	0 (0.0%)	0 (0.0%)
	Maintenance dose	75 (100.0%)	75 (100.0%)
Indication	Neoadjuvant treatment: HER2-positive, locally advanced, inflammatory or early-stage breast cancer (either greater than 2 cm in diameter or node positive)	1 (1.3%)	0 (0.0%)
	Adjuvant treatment: HER2-positive early breast cancer at high risk of recurrence	17 (22.7%)	9 (12.0%)
	HER2-positive metastatic or locally recurrent unresectable breast cancer, who have not received previous anti-HER2 therapy or chemotherapy for their metastatic disease	57 (76.0%)	66 (88.0%)

HER2: Human epidermal growth factor receptor 2.

### Primary end points

The first primary end point, patient length of stay during treatment, was significantly shorter in the SC arm as compared with the IV arm by a mean difference of 190.9 ± 13.6 min saved per patient per visit (95% CI: 163.9, 217.9). The second co-primary end point, pharmacist preparation time, was also significantly shorter with the SC formulation (median 8 min [Q1: 4; Q3: 15]) compared with IV (median 13 min [Q1: 6; Q3: 28]; p = 0.006; Table 3).

**Table 3.** Duration of procedures.

Variable		Fixed-dose combination of pertuzumab and trastuzumab for subcutaneous injection	Pertuzumab and trastuzumab for intravenous infusion	Difference (95% CI)	p-value
		n = 75	n = 75		
Duration of nurse administration of treatment (minutes)	Mean ± SD	5.0 ± 0.4	109.7 ± 27.7	104.7 ± 3.2	<0.001^†^
	Median (Q1 – Q3)	5 (5–5)	123 (80–130)	(98.3, 111.0)	
	Min – Max	5–8	60–164		
Duration of post-treatment monitoring (minutes)	Mean ± SD	45.2 ± 37.0	44.8 ± 36.7	-0.4 ± 6.0	0.983
	Median (Q1 – Q3)	36 (20–64)	35 (22–56)	(-12.3, 11.5)	
	Min – Max	0–219	6–230		
Chair occupancy duration (minutes)	Mean ± SD	50.2 ± 37.0	154.8 ± 37.8	104.5 ± 6.1	<0.001^†^
	Median (Q1 – Q3)	41 (25–69)	153 (130–168)	(92.5, 116.6)	
	Min – Max	5–224	85–300		
Pharmacist preparation duration (minutes)	Mean ± SD	13.3 ± 15.5	22.3 ± 25.2	9.0 ± 3.4	0.006^†^
	Median (Q1 – Q3)	8 (4–15)	13 (6–28)	(2.2, 15.7)	
	Min – Max	1–74	2–138		
Patients' length of stay during treatment (minutes)	Mean ± SD	167.3 ± 56.4	358.2 ± 103.8	190.9 ± 13.6	<0.001^†^
	Median (Q1 – Q3)	172 (129–194)	339 (280–444)	(163.9, 217.9)	
	Min – Max	70–401	169–622		

† Significant difference in duration between the two study arms with a two-sided significance level of 5%.

‡ The duration in the infusion was compared with 0 using Wilcoxon signed rank test.

SD: Standard deviation.

### Secondary end points

Time savings were translated into operational and labor cost savings. The mean nurse administration time saved per visit was 104.7 ± 3.2 min (Table 3), which corresponds to a direct labor-cost saving ranging between 87.25 AED (≈ 23.8 USD) and 174.5 AED (≈ 47.6 USD), depending on whether the nurse hourly rate was set at 50 or 100 AED (Table 4). Assuming a 100% switching rate of all IV patients to the SC formulation for the study cohort (1350 annual visits), this equated to a total of 2355.75 saved nursing hours per year (equivalent to ~294.5 full working days based on an 8 h workday; Table 4). In terms of pharmacist preparation time, the median time saved was 5 min (SC: 8 min [Q1: 4; Q3: 15] vs IV: 13 min [Q1: 6; Q3: 28]) (Table 3), corresponding to a labor saving of 8.3 to 12.5 AED (≈ 2.3 to 3.4 USD) per visit, considering a pharmacist hourly labor rate ranging between 100 and 150 AED. Under the same 100% switching assumption, the institution saved 112.5 pharmacists’ hours every year (equating to ~14 full working days of pharmacy productivity). When extrapolated to the study cohort, these per-visit savings correspond to an annual indirect cost saving ranging between 129,037 and 252,450 AED (≈35,160 to 68,787 USD), reflecting a total of 2468.25 clinical hours saved annually across nursing and pharmacy services (equivalent to ~308.5 full working days; Table 4).

**Table 4.** Annual indirect labor cost savings calculation.

Component	Time saved per visit (min)	Time saved per visit (hours)	Total annual hours saved (1350 visits)	Total working days saved (8 h day)	Hourly rate range (AED)	Formula for cost saving per visit	Formula for cost saving (Annual, i.e., 1350 visits)	Cost saving per visit (AED)	Annual saving (1350 visits) (AED)	Annual saving (1350 visits) (USD)
Nurse administration	104.7 min	104.7/60 = 1.745 h	104.7 min × 1350 visits/60 = 2355.75 h	2355.75/8 = 294.5 days	50–100	1.745 h × hourly rate	2355.75 h × hourly rate	87.25–174.5	117,787–235,575	32,094–64,189
Pharmacist preparation time	5 min	5/60 = 0.083 h	5 min × 1350 visits/60 = 112.5 h	112.5/8 = 14 days	100–150	0.083 h × hourly rate	112.5 h × hourly rate	8.3–12.5	11,250–16,875	3065–4598
Total	109.7 min	1.828 h	2468.25 h	2468.25/8 = 308.5 days	—	Nurse saving + pharmacist saving		95.55–187.0	129,037–252,450	35,160–68,787

Annual savings were calculated based on a cohort of 75 patients undergoing 18 treatment cycles per year, corresponding to 1350 visits annually. Saved minutes were converted into hours before multiplying by assumed hourly rates. These figures are potential saved costs based on assumed hourly rates, as actual institutional rates are confidential. USD equivalents are calculated at the fixed conversion rate of 1 USD = 3.67 AED.

Additional measures showed that the duration of nurse administration of treatment was significantly shorter in the SC arm as compared with the IV arm (5.0 ± 0.4 vs 109.7 ± 27.7 min; p < 0.001). Duration of post-treatment monitoring was similar between the two study arms (45.2 ± 37.0 min in SC arm vs 44.8 ± 36.7 min in IV arm; p = 0.983). Parallel to labor savings, chair occupancy duration was also significantly shorter among patients in the SC arm as compared with the IV arm (50.2 ± 37.0 vs 154.8 ± 37.8 min; p < 0.001; Table 3).

## Discussion

The objective of the study was to measure time saving and indirect cost saving associated with using PH FDC SC compared with IV infusion of pertuzumab and trastuzumab in patients with HER2-positive breast cancer. The SC FDC was associated with a significantly shorter duration of treatment administration by nurses and patient length of stay, as well as shorter pharmacist preparation time with the SC formulation. This was in line with a substantial body of evidence demonstrating the time-saving aspect of PH FDC SC [8]. In addition to reduced administration time, the SC formulation has offered several advantages including more patient convenience, improved health-related quality of life, optimized healthcare resource utilization (HCRU) and minimizing costs of cancer treatments [8,12–14].

In the present study, optimizing HCRU was achieved through reduced patient chair occupancy and saving healthcare providers’ time (nurses and pharmacists). Similar findings were demonstrated in the time-and-motion substudy of the PrefHer trial as well as in other studies [8,9,15]. To accurately understand the operational impact, it is important to distinguish between ‘active nursing workload’ and total ‘chair occupancy duration’. Even though a nurse only spends a few minutes active at the start and end of an IV, the patient continues to occupy a treatment chair for the entire duration of the infusion. Specifically, the duration of nurse administration of treatment was significantly shorter in the SC arm (5.0 ± 0.4 vs 109.7 ± 27.7 min; p < 0.001), representing a 95.4% reduction in active nursing workload. The mean nurse time saved per cycle (104.7 min) represents a comprehensive reduction in workload across the entire patient encounter (encompassing preparation, administration and monitoring). Assuming a 100% switching rate of all IV patients to the SC formulation for the study cohort of 75 patients undergoing 18 cycles per year (1350 annual visits), this would result in a total of 2355.75 annual nursing hours saved (equivalent approximately to 294.5 full working days based on an 8 h workday). The true institutional advantage is that the SC formulation frees up chair capacity much faster and clears those chairs for other patients almost immediately. Furthermore, total chair occupancy was significantly shorter in the SC arm (50.2 ± 37.0 vs 154.8 ± 37.8 min; p < 0.001), reclaiming 104.5 min (~1.75 h) of physical chair capacity per patient visit. For the annual cohort of 1350 visits, this represents 2351.25 reclaimed chair hours. Based on an average chemotherapy infusion duration of 1.5 h as per the oncology pharmacy manual of the institution, this saved chair capacity would allow the center to accommodate approximately 1567 additional chemotherapy infusions annually (calculated as 2351.25 h/1.5 h). This significant increase in throughput equates to roughly 8 ± 2 additional daily patient appointments, providing actionable evidence for hospital administrators to optimize existing treatment space and reduce patient waiting lists in the oncology service. For pharmacist preparation time, time spent by the pharmacist to prepare three IV infusions was also the same time they will spend to prepare five SC injections. Applying the same 100% switching rate, the institution would save 112.5 pharmacist hours every year (~14 full working days of pharmacy productivity).

Consequently, enhanced patient throughput and shorter waiting lists would be expected. Alternatively, time saved by healthcare providers could be redirected to other patient care activities, thereby improving overall staff efficiency in treatment centers and optimizing HCRU within the treatment center. The difference between IV and SC formulations represents the closest estimation of annual indirect cost savings. In this study, the per-visit savings in nurse administration (87.25–174.5 AED, equivalent to 23.8–47.6 USD) and pharmacist preparation (8.3–12.5 AED, equivalent to 2.3–3.4 USD) were multiplied by the annual cohort of 75 patients undergoing 18 cycles (1350 visits). This extrapolation yielded nurse administration time savings of 117,787 to 235,575 AED (equivalent to 32,094 to 64,189 USD) annually, while pharmacist preparation time savings corresponded to 11,250 to 16,875 AED (equivalent to 3065 to 4598 USD) annually. Together, these extrapolated figures reflect a total potential annual indirect cost saving of 129,037 to 252,450 AED (equivalent to 35,160 to 68,787 USD), derived from a total of 2468.25 clinical hours recovered annually across nursing and pharmacy services. These are potential savings based on assumed hourly rates, as actual institutional rates are confidential. According to internal analyses conducted in 2022 and 2023 at Tawam Hospital (preliminary internal audit), eliminating drug preparation and infusion time because of switching from IV to SC formulation resulted in substantial labor savings: 99,855 min (≈1664 h) in 2022 and 105,650 min (≈1761 h) in 2023 from combined pharmacy and nursing time. While these internal figures confirm the overall trend of institutional efficiency, they were derived using a different calculation methodology and sample size than the current study; nonetheless, both datasets underscore the consistent operational gains achieved through SC administration. These figures highlight the operational efficiency gains in terms of labor hours saved when switching from IV to SC administration, without reference to drug acquisition cost differences or individual drug costs, which remain confidential.

Nondrug costs were reduced after switching from IV to SC formulation. A cost-minimization analysis reported similar findings; PH FDC SC was estimated to reduce nondrug costs by 73–80% in Western Europe, and 75% in the USA [16]. The findings at Tawam Hospital mirror these international trends; as the locally observed 95.4% reduction in active nursing workload and significant chair-time recovery (104.5 min per visit) confirms the massive efficiency gains seen in hospitals worldwide. This reduced nondrug cost was mainly in terms of savings in patient chair time and to a lesser extent patients' productivity loss. Moreover, similar analyses reported that PH FDC SC was associated with significant reduction in the cost of nondrug consumables [9,17]. PH FDC SC contributes to considerable healthcare cost savings, consequently, reducing the overall financial burden on healthcare systems. Such reduction would contribute to the long-term sustainability of the healthcare spending, while still providing a safe and effective therapy.

From a patient’s perspective, PH FDC SC was preferred over the conventional IV formulation [4,11]. This preference was driven by the shorter administration time, the convenient and comfortable administration process, and positive impact on patients’ quality of life [4,8,11]. While these benefits have been well-documented for other SC oncology formulations, they are particularly relevant to PH FDC SC, which further optimizes the treatment process by combining pertuzumab and trastuzumab into a single injection. In terms of patient convenience and preference, the concept of flexible care has been an attractive alternative to traditional therapy administration in hospital settings. By taking advantage of these established SC formulations in cancer therapy, flexible care allows patients to receive their cancer therapy at home or in primary care settings. Consequently, patients would be able to maintain their regular daily activities regularly, while reducing healthcare system costs in terms of healthcare providers time and hospitals occupancy. Present findings suggest this move toward flexible care is a perfect fit for PH FDC SC, mainly because the drug is so much faster and easier to give than traditional IV infusions.

Notably, while prescribing information recommends a post-treatment monitoring period of only 15–30 min for the SC formulation [6], a duration of 45 min was observed in the current real-world study. This discrepancy suggests that actual clinical practice at the center currently includes additional observation time beyond the minimum requirements, likely due to institutional workflows, concurrent nursing assessments, or the initial implementation phase where nursing staff exercised additional caution while becoming familiar with the new FDC formulation. In real-world settings, recorded monitoring times often deviate from strict guidelines due to practical variables such as coordinated patient discharge, transport logistics or administrative documentation. By strictly adhering to the 15 min observation protocol once the formulation is fully integrated into routine practice, the center could realize even greater time-saving benefits. Our data confirms that monitoring time savings were indeed captured and contributed to the total mean nurse time saved, though the magnitude of the difference reflects real-world institutional practice at Tawam Hospital rather than a controlled trial environment. Additionally, the time needed by the pharmacist to prepare a SC dose is estimated at 5 min, compared with the 8 min recorded in the present study. This 3 min variance is largely attributed to the required period for the FDC formulation to reach room temperature after being removed from refrigeration, as well as institutional logistical steps such as order verification, batching and internal delivery, rather than a lack of efficiency in the technical preparation itself. This indicates that the time-saving potential with the SC formulation is still to be fully achieved by minimizing both the pharmacist preparation time of SC injection and the post-treatment monitoring to match what is recommended in the summary of product characteristics. Aligning local workflows with these 5 min preparation and 15 min monitoring targets will be reflected in relevant cost saving greater than what was achieved so far. The data also did not take into consideration the direct cost saving from reduced use of consumables (IV infusion bag, infusion line and catheter are needed for the IV formulation vs a 10–15 ml syringe, a transfer needle and a 25–27G injection needle needed for the SC). Factoring in the differential cost of consumables between formulations would further increase the estimated economic benefit of SC administration.

The first limitation in this study is that it was conducted in a single center. Healthcare practices and resources may not represent those in other hospitals across the UAE; thus, the findings may not be generalizable to other hospitals where resource allocation, clinical practice, and treatment processes differ. However, the alignment of our data with global findings suggests these efficiencies are achievable in similar settings. Second, time efficiency observed in this study may be influenced by the specific workflow and infrastructure of the study site. Third, cost savings were calculated based on local costs of the center, which may vary widely between institutions and countries. In addition, the cost of medical supplies/consumables (e.g., IV infusion bags and lines for the IV arm vs syringes and needles for the SC arm) were not included in the analysis. Given that SC administration significantly reduces the volume and complexity of required materials, the current figures likely represent a conservative underestimation of the true total economic benefit of the SC formulation.

In conclusion, PH FDC SC was associated with significant time savings for both patients and healthcare providers, including shorter nurse administration and pharmacist preparation times. Switching from IV to SC administration reclaimed a total of 2468.25 clinical hours annually (equivalent to ~308.5 full working days) across nursing and pharmacy services for a cohort of 75 patients undergoing 18 cycles (1350 visits). These time savings translated into notable indirect cost reductions ranging between 129,037 AED and 252,450 AED (equivalent to 35,160 to 68,787 USD) annually, derived from nurse and pharmacist labor time and assumed hourly rates. Also, this transition provided indirect benefits such as shorter patient stays, reduced chair occupancy and increased chair capacity, leading to improved workflow efficiency. These findings highlight the potential of PH FDC SC to improve operational efficiency, optimize resource utilization and enhance patient experience within cancer treatment centers. Further multicenter studies are needed to confirm the generalizability of these results across different healthcare settings.

## Summary points

- The subcutaneous (SC) formulation of a new fixed-dose combination (FDC) of pertuzumab, trastuzumab and hyaluronidase–zzxf was approved by the US FDA on 29 June 2020.
- Studies have shown that the PH FDC SC is as safe and effective as the intravenous (IV) formulation.
- The SC formulation of PH FDC SC allows patients to receive treatment in 5–8 min compared with approximately 30–90 min with the IV formulation.
- The objective of this observational retrospective study was to measure time saving associated with using PH FDC SC compared with pertuzumab and trastuzumab for IV infusion in patients with HER2-positive breast cancer.
- Fifty patients with three visits per patient were included in the final analysis.
- Patients’ length of stay was significantly shorter in the SC arm by a mean difference of 190.9 ± 13.6 min saved per patient per visit (95% CI: 163.9, 217.9).
- Median pharmacist preparation time was significantly shorter (8 min [Q1: 4; Q3: 15]; p = 0.006) with the SC formulation compared with the IV formulation (13 [Q1: 6; Q3: 28]).
- The transition to SC administration significantly reduced chair occupancy duration by a mean of 104.5 min per visit; this reclaimed chair capacity would allow the center to accommodate approximately 1567 additional chemotherapy infusions annually.
- PH FDC SC was associated with significant time savings for both patients and healthcare providers. Assuming a 100% switching rate for a cohort of 75 patients (1350 annual visits), the transition to SC would unlock 2355.75 nursing hours and 112.5 pharmacist hours annually.
- These time savings (2468.25 total hours, equivalent to ~308.5 full working days) translated into potential indirect cost reductions of 129,037 AED to 252,450 AED (equivalent to 35,160 to 68,787 USD) annually, based on assumed hourly labor rates for healthcare providers.
